# Risk Evaluation for Coating Thickness Conformity Assessment

**DOI:** 10.3390/ma16020758

**Published:** 2023-01-12

**Authors:** Dubravka Božić, Marina Samardžija, Marin Kurtela, Zdenka Keran, Biserka Runje

**Affiliations:** 1 Department of Quality, Faculty of Mechanical Engineering and Naval Architecture, University of Zagreb, 10000 Zagreb, Croatia; 2 Department of Chemistry, Faculty of Mining-Geology-Petroleum Engineering, University of Zagreb, 10000 Zagreb, Croatia; 3 Department of Welded Structures, Faculty of Mechanical Engineering and Naval Architecture, University of Zagreb, 10000 Zagreb, Croatia; 4 Department of Technology, Faculty of Mechanical Engineering and Naval Architecture, University of Zagreb, 10000 Zagreb, Croatia

**Keywords:** global consumer’s risk, global producer’s risk, conformity assessment, measurement uncertainty, gray cast iron, epoxy coating

## Abstract

This paper presents the conformity assessment process of the epoxy coating thickness applied on water pipes made of gray cast iron with the specifications given for this kind of coating appliance. An epoxy coating was applied to prevent a special form of corrosion called the graphitization of cast iron. In order for the pipe to withstand its designed service life, it is necessary to ensure the required thickness of the applied coating. In accordance with the EN 877 norm, the thickness of the epoxy coating on the pipes for the projected corrosiveness of the environment C4 and the durability of 20 years is at least 70 μm and this indicates the required accuracy of the product. To achieve the desired product quality, statistical control of the coating application process was carried out and the impact of uncertainty associated with the measurement result was analyzed. Considering the quality of the coating application process and the quality of the measuring system, and to ensure the quality of products and to reduce consumer risk, the optimal thickness of the coating was determined.

## 1. Introduction

A conformity assessment process is any activity undertaken to determine whether a product, process, or system meets the requirements of certain standards or meets predefined requirements [1].

In the process of assessing the conformity of products, it is necessary to compare the measured value with the predefined product specification to confirm its compliance. This paper carries out the process of assessing conformity with the specification prescribed for the thickness of the epoxy coating on water pipes made of gray cast iron. Gray cast iron is often used to make water pipes dug into the soil because of its flexibility, good cast ability, low-cost [2], excellent mechanical properties, and desirable castability [3]. In addition, it is used for its resistance to moderately high and usually variable water pressures [4]. Unfortunately, the damage of gray cast iron at the exterior parts through electrochemical corrosion has been the predominant restricting mechanism against enhancing its life span [5]. Gray cast iron begins to lose its properties during exposure of the metal surface to an aggressive environment, and a special form of corrosion occurs which is called the graphitization of cast iron [6]. One way to solve this problem is to protect the metal surface by insulating it from the environment. For this purpose, the choice of an appropriate coating system shall be applied [7,8]. Among various metal anticorrosion methods, organic coating is one of the most economical, effective, and common methods [9]. Simple application, good adhesion to metal substrates, increased strength and hardness, high resistance to chemicals and other corrosive media, and low cost make epoxy coating a key method for water pipes protecting [10,11]. These organic films, including polyurethane, polyamide, polyester, resin, and epoxy, play a crucial role as a barrier layer to avoid the transportation of corrosive species, such as chloride and hydroxyl ions, oxygen, water, pollutants, pigments, and other substance [12]. In order for the applied coating to withstand the designed service life of the pipe, it is necessary to ensure the required thickness of the coating [13]. In accordance with EN 877 [14], the thickness of the epoxy coating on the pipes for engineered environmental corrosivity C4 and the durability of 20 years is at least 70 μm [15]. The specified limit in the conformity assessment process is prescribed and indicates the required accuracy for the product. During exploitation, damage to the epoxy coating occurs. As a result of the damage, bubbles, micropores, and microcracks are formed [16], and it is necessary to adjust the thickness of the coating so that the structure will withstand the designed service life. The thickness of the dry layer of the coating is a very important parameter for determining the quality of protection of the metal surface. The greater the thickness, the greater the barrier between the metal and the environmental medium, and the more difficult it is for the substrate to corrode. Coatings that are too thin are not durable and coatings that are too thick are expensive and often poorly adhere to the substrate due to internal tensions, so for economic reasons, it is important to reliably know the minimum values of coating thickness. Organic coating degradation is a worldwide phenomenon that causes physical changes to the coating and costly repairs [17]. The thickness of the coating can increase significantly after immersion in liquid due to the formation of bubbles on the surface of the coating. However, if there are no visible bubbles on the surface of the coating, the thickness of organic coatings decreases after immersion [18]. The application of the coating is carried out by immersing the pipe in an epoxy coating, then it is heat treated at 180° C for 45 min. The preparation of the metal surface before the application of the epoxy coating must be implemented in accordance with HRN EN ISO 8501-1 [19]. This paper presents a procedure for calculating a specific and global risk for assessing conformity with the specification for the epoxy coating thickness on water pipes made of gray cast iron. Specific risk is defined as the probability of a wrong decision for a particular product, and global risk is defined as the probability of a wrong decision based on future measurements [1]. The risk of acceptance of a non-conforming product (the so-called consumer risk) and the risk of rejection of a conforming product (the risk of the producer) are considered. Both types of risk are calculated using the Bayesian framework. In the conformity assessment process, the measurement uncertainty of the results plays an important role. The measurement result is complete if it contains not only the measured value but also the associated measurement uncertainty. Measurement uncertainty is a parameter associated with a measurement result that describes the dispersion of the values that could reasonably be attributed to a measurand [20]. Measurement uncertainty means doubts about the validity of the measurement result and represents the quality of the measurement result [21]. In order to achieve the desired product quality, statistical control of the coating application process was carried out and the impact of uncertainty associated with the measurement result was analyzed. Considering the quality of the coating application process and the quality of the measuring system, and to ensure the quality of products and to reduce consumer risk, the optimal thickness of the coating was determined.

## 2. Materials and Methods

The process of applying epoxy coating on the outer side of a pipe is followed by an X-bar control chart. Through statistical control of the process carried out on a sample of 75 pipes, the thickness of the coating was ascertained as well as the standard deviation for the straight and junction portions of the pipe. The thickness of the coating significantly affects the functionality of the coating and also affects the functionality of the pipe. Therefore, the techniques for determining the thickness of the coating are extremely important [22]. The measurements on the straight and junction portion of the pipe have been carried out in three different measurement points via the application of the Elcometer^®^ 456 device (Elcometer Limited, Edge Lane, 136 Manchester, UK). These probes for coating thickness gauge can measure ferrous and non-ferrous applications with automatic substrate detection. Measurements can be performed on smooth, rough, thin, and curved surfaces in accordance with national and international standards. Before starting the measurement of the dry coating film thickness, the device was calibrated using a set of calibration foils (Elcometer 990 Calibration Foils, Manchester, UK). In our measurement case, the device was calibrated to a thickness of 75.1 μm using the combined calibration foils or ‘shims’ with an uncoated substrate (Zero Test Plate). The results of the measurement and the pertaining standard uncertainty measures are provided in Table 1.

In accordance with norm EN 877, the epoxy coating thickness for pipes must last for 20 years and be at least 70 µm thick if it is to be in accordance with the designed corrosiveness of the environment C4.

The purpose of this research is to evaluate consumer and manufacturer risk and determine the optimal coating thickness considering the standard deviation value $u_{0}$ and the standard measurement uncertainty $u_{m}$ which characterize the thickness application process as well as the quality of the measurement system. The $u_{0}$ standard deviations derived from the production process indicate a significant dispersion of the results generated within the coating application process. A significantly greater dispersion of the results was achieved on the junction portion of the pipe which points to difficulties in applying coating on this section of the pipe.

Due to the large dispersion of the results in the process of the application of the coating, the producers apply a much greater coating quantity of 92 µm and 170 µm depending on which portion of the pipe they are working on.

Besides the dispersion caused by the production process, this paper also analyzes the impact of the dispersion of the results caused by the application of the measurement system. The quality of the measurement system is described by the amount of standard measurement uncertainty $u_{m}$.

In the process of the coating thickness measurement, there are numerous values that significantly impact the uncertainty of measurement um. The main sources that contribute to the measurement uncertainty of the coating thickness measurement result are the instrument used in the measurement process, the standard for instrument fine-tuning, the repeatability and the reproducibility of the instrument positioning, the geometry of the surface of the measured subject (the curve of the surface and the deviations of the flatness), and the impact of the temperature [23]. By applying the design of the experiment and the scientific judgment of all of the available information about the possible variability in the input quantity, a mathematical model, which describes the measuring system, was obtained. For the successful evaluation of the uncertainty of measurement, the most important thing is the close connection of the mathematical model of the measuring system and the measurement itself or completing the experiment in a manner so that all of the significant impacts on the measured uncertainty are varied [23].

The measurement uncertainty is estimated via the application of the GUM method (guide to the expression of uncertainty in measurement) [20] and the MCS (Monte Carlo) method [24]. While the GUM method assumes normal distribution of the output value, the MCS method yielded experimental distribution of the output value that may more or less match the assumed normal distribution [25]. The form of the experimental curve will depend primarily on the probability density function of the most significant input value [26,27,28].

In order to verify the influence of the functional relationships of the input quantities on the output quantity, the MCS method was implemented. The function of the density probability of the coating thickness was obtained by the convolution of the input quantities using 100,000 simulations.

The evaluation of consumer risk and producer risk was carried out for data which were characterized by the production processes (Table 1) with the condition of the minimal necessary thickness of the coating T_L_ = 70 µm and T_L_ = 80 µm. In order to analyze the impact of the quality of the measurement system on producer and consumer risk, an analysis was also carried out for the standard measurement uncertainties $u_{m}$ = 2 µm, for the straight part of the pipe, and for the standard measurement uncertainties $u_{m}\;$= 4 µm for the pipe junction.

The risk assessment for consumers and producers is conducted via the methodology described in the reference document JCGM 106:2012 [1]. According to this document, the consumer’s risk is defined as the risk of accepting a non-conforming measurement. The producer’s risk is defined as a risk of rejecting a conforming measurement. This verification process is known as the conformity assessment rule.

When calculating the producer’s and the consumer’s risk, the so-called Bayes approach is applied [29]. This approach combines two sources of information [30,31]. The first source of information relates to the item of interest. In this case, this is the coating thickness. This information is described by the random variable Y which can take the values η. The probability density function (PDF) associated with random variable Y usually is called *prior* and it is denoted by g_0_ (η). Considering that the coating thickness is always strictly greater than 0, for the value of the argument of prior distribution holds that η > 0. For modelling of the prior distribution, two quantities associated with the random variable Y are used: best estimate ӯ and standard deviation $u_{0}.$ In accordance with [1], in this research, the gamma distribution Γ(η; α, λ) given by the following formula was chosen as the prior:

(1) $$\Gamma \left(\eta ;\;\alpha ,\;\lambda \right)=\frac{\lambda^{\alpha }}{\Gamma \left(\alpha \right)}\eta^{\alpha -1}e^{-\lambda \eta }.$$

Parameters α and λ, for the gamma distribution, are calculated according to the following formulas:

(2) $$\left\{\begin{array}{c} \lambda =\frac{\;ӯ}{u_{0}{}^{2}}\\ \alpha =\frac{{\;ӯ}^{2}}{u_{0}{}^{2}} \end{array}\right.$$

The second source of information is data assigned to the measurements described by the random variable Y_m_. The value of the measured quantity is denoted by $\eta_{m},$ and the associated standard measurement uncertainty is denoted by $u_{m}$ [20]. Those data are modelled via the likelihood function for the normal distribution denoted by $h\left(\eta_{m}|\eta \right)$ which is given by the following formula:

(3) $$h\left(\eta_{m}|\eta \right)=\frac{1}{u_{m}\sqrt{2\pi }}\exp\left[-\frac{1}{2}{\left(\frac{\eta_{m}-\eta }{u_{m}}\right)}^{2}\right].$$

According to the Bayesian rule, by combining the prior and the likelihood function, the expression for the posterior distribution can be derived, and it is given as:

(4) $$g\left(\eta |\eta_{m}\right)={\mathrm{Cg}}_{0}\left(\eta \right)h\left(\eta_{m}|\eta \right),$$

where the constant C is the normalization constant so that $\int_{-\infty }^{\infty }g\left(\eta |{\;\eta }_{m}\right)d\eta =1$.

Two important intervals are necessary in order to determine the risk to producers and the risk to consumers. Tolerance interval [T_L_, T_U_] is interval of permissible values of a item of interst, given by standard for given products. Labels T_L_ and T_U_ are lower and upper tolerance limit, respectively.

In this research, two tolerance intervals were observed. The tolerance interval with the lower boundary T_L_ = 70 μm whose upper limit theoretically is placed in infinity, and the tolerance interval with the lower limit T_L_ = 80 μm with the upper limit placed also at infinity. The conformance probability that the item of interest is within the tolerance interval can be calculated as:

(5) $$p_{c}=\int_{T_{L}}^{T_{U}}g\left(\eta |\eta_{m}\right)d\eta$$

**Figure 1:**
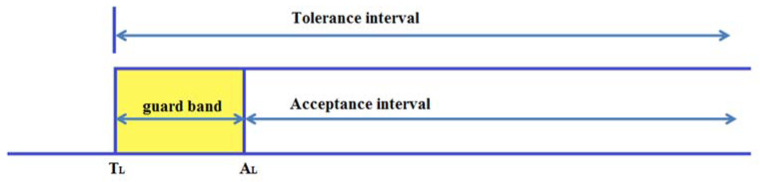
Acceptance and tolerance interval [32].

The second important interval is the acceptance interval [A_L_, A_U_], where A_L_ and A_U_ are the lower and upper limits of the acceptance interval, respectively. The acceptance interval and the tolerance interval can be in a variety of relationships with one another [32]. In order to minimize the consumer’s risk, in this research the acceptance interval has been placed within the tolerance interval, Figure 1.

These intervals are separated by a guard band of the width $w=2{\mathrm{ru}}_{m}.$ Now, the lower limit of the acceptance interval for a given $u_{m}$ can be calculated from:

(6) $$A_{L}=T_{L}+2{\mathrm{ru}}_{m}$$

The multiplier r is in the range from −1 to 1.

If the true value Y of the item of interest is outside the tolerance interval and the measured value Y_m_ is within the acceptance interval, the global consumer’s risk R_C_ can be calculated as follows:

(7) $$R_{C}=\int_{-\infty }^{T_{L}}\int_{A_{L}}^{A_{U}}g_{0}\left(\eta \right)h\left(\eta_{m}|\eta \right){d\eta }_{m}d\eta +\int_{T_{U}}^{\infty }\int_{A_{L}}^{A_{U}}g_{0}\left(\eta \right)h\left(\eta_{m}|\eta \right){d\eta }_{m}d\eta$$

If the true value Y of the item of the interest is within the tolerance interval and the measured values Y_m_ is outside the acceptance interval, global producer’s risk R_P_ can be calculated from the following formula:

(8) $$R_{P}=\int_{-\infty }^{A_{L}}\int_{T_{L}}^{T_{U}}g_{0}\left(\eta \right)h\left(\eta_{m}|\eta \right){d\eta }_{m}d\eta +\int_{A_{U}}^{\infty }\int_{T_{L}}^{T_{U}}g_{0}\left(\eta \right)h\left(\eta_{m}|\eta \right){d\eta }_{m}d\eta .$$

By placing the expression for prior distribution and the likelihood function in Formulas (7) and (8) and by introducing the substitution

(9) $$z=\frac{\eta_{m}-\eta }{u_{m}},$$

with the assumption that the upper levels of the acceptance interval and the tolerance interval are placed at infinity, the expressions for consumer’s risk and producer’s risk are given in a final form as:

(10) $$\mathrm{Rc}=\int_{-\infty }^{T_{L}}\left(1-\phi (\frac{A_{L}-\eta }{u_{m}})\right)g_{0}\left(\eta \right)d\eta ,\;\frac{A_{L}-\eta }{u_{m}}>0$$

(11) $$R_{P}=\int_{T_{L}}^{\infty }\left(\;\phi (\frac{A_{L}-\eta }{u_{m}})\right)g_{0}\left(\eta \right)d\eta ,\frac{A_{L}-\eta }{u_{m}}>0$$

The designations $\phi \left((A_{L}-\eta \right)/u_{m})$ in Formulas (10) and (11) relate to the cumulative distribution function (CDF) for the standard normal distribution function with the variable $(A_{L}-\eta )/u_{m}.$

## 3. Models and Results

### 3.1. Straight, Outside Section of the Pipe

Four models for the thickness of the epoxy coating on the outside, the straight section of the pipe, were observed. According to the data obtained from the production process, the best estimation of the epoxy coating thickness is ӯ = 92 μm, with a standard deviation of $u_{0}=16$ μm. Lower limits of tolerance intervals of 70 μm and 80 μm, set according with the EN 877 norm, were observed for models whose standard measurement uncertainty was $u_{m}=2\;$μm and $u_{m}=4\;$μm, respectively. For the purpose of simplicity, in the further text, we introduce labels for the observed models $M_{i}\left(T_{L},\;ӯ,{\;u}_{0},{\;u}_{m}\right)$. The ordinal number of the models is marked with *i*. In addition to the labels introduced in this way, the following models for the thickness of the epoxy coating on the straight, outer side of the pipe were observed: $M_{1}\left(70,\;92,\;16,\;2\right),$ $M_{2}\left(80,\;92,\;16,\;2\right),$ $M_{3}\left(70,\;92,\;16,\;4\right)$ and model $M_{4}\left(80,\;92,\;16,\;4\right)$.

**Figure 2:**
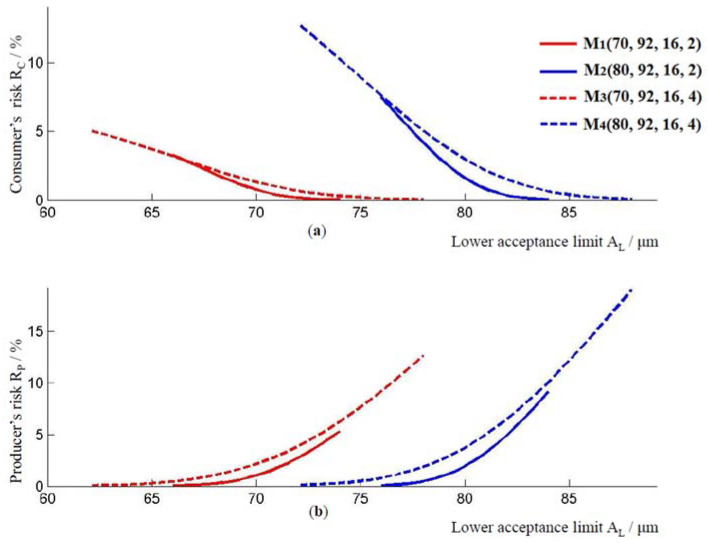
(**a**) Consumer’s risk and (**b**) producer’s risk, for models $M_{1},{\;M}_{2},{\;M}_{3}\text{ and}{\;M}_{4}$.

Since multiplier *r* is in the range from −1 to 1, the method for risk calculation, according to (6) allows risk assessment for coating thickness in the range of [W_L_, W_U_]. The lower limit of the range for the coating thickness, W_L_, is obtained for r = −1, and the upper limit of the range for the coating thickness, W_U_, is obtained for r = 1. The upper limit of the coating thickness W_U_ is also the lower limit of the acceptance interval, i.e., W_U_ = A_L_ is valid. The coating thickness ranges for models $M_{1},{\;M}_{2},{\;M}_{3}$, and $M_{4}$ are [66, 74] μm, [76, 84] μm, [62, 78] μm, and [72, 88] μm, respectively. In all of the models, the highest consumer risk and the lowest producer risk are achieved when the coating thickness is equal to W_L_. The lowest consumer risk and the greatest risk to the producer are achieved when the coating thickness is equal to W_U_, i.e., at the lower limit of the acceptance interval A_L_, Figure 2. The lower limits of the acceptance interval for models $M_{1},{\;M}_{2},{\;M}_{3},$ and $M_{4}$ are 74 μm, 84 μm, 78 μm, and 88 μm, respectively. For these values of the lower limits, the following consumer’s and producer’s risks were obtained. For model $M_{1}$, when A_L_ = 74 μm, the consumer’s risk holds R_C_ = 0.018%, and the producer’s risk holds R_P_ = 5.287%. For model $M_{2},$ when the lower limit of the acceptance interval is A_L_ = 84 μm, the consumer’s risk holds R_C_ = 0.038%, and the producer’s risk holds R_P_ = 9.145%. For model $M_{3}$, for A_L_ = 78 μm, the consumer’s risk holds R_C_ = 0.032%, and the producer’s risk holds R_P_ = 12.663%. For model $M_{4}$ when A_L_ = 78 μm, the consumer’s risk holds R_C_ = 0.069%, and the producer’s risk holds R_P_ = 19.025%.

On the other hand, Figure 2 simultaneously shows the impact of the standard measurement uncertainty $u_{m}$ on consumer and producer risk. Models $M_{1}$ and $M_{3}$ differ only in the standard measurement uncertainty. Model $M_{3}$ has a higher standard measurement uncertainty $u_{m}$ than model $M_{1}$. All of the other parameters are the same for both models. When these models are compared, it is obvious that the model with the higher measurement uncertainty has both a higher consumer’s risk and a higher producer’s risk, in the range provided by the norm, i.e., for $T_{L}>70$ μm. The same applies to models $M_{2}$ and $M_{4}$, for $T_{L}>80$ μm. Model $M_{4}$ has a higher measurement uncertainty compared to the $M_{2}$ model, and a higher producer’s and consumer’s risk compared to the $M_{2}$ model.

The mean value of the interval [W_L_, W_U_] = [W_L_, A_L_] is the lower limit of the T_L_ tolerance interval. This value divides the interval [W_L_, A_L_] into two intervals, interval [W_L_, T_L_] and interval [T_L_, A_L_]. Interval [W_L_, T_L_] is not the interval of permitted values for the thickness of the epoxy coating, but due to the measured uncertainty, it may happen that the consumer is supplied with such a product. One of the goals of this paper is to reduce the possibility of the delivery of pipes in which the thickness of the epoxy coating is within the interval [W_L_, A_L_]. Interval [T_L_, A_L_] is the guard band interval. Outside this interval, in the area from A_L_ to infinity, the consumer’s risk values fall, while the values for producer’s risk rise.

The conformance probability for models $M_{1}$ and $M_{3,}$ for T_L_ = 70 μm are the same and equivalent to 0.9251. This means that there is 92.51% of conforming coatings and 7.49% of non-conforming coatings. The percentage of falsely rejected products is obtained by subtracting the producer’s risk value from the value for conformance probability. The percent of falsely accepted coatings is obtained by subtracting the consumer’s risk from the percentage of non-conforming coatings, Table 2.

By simply multiplying these percentages with the number of pieces of pipe for which the calculation is made, the number of accepted, falsely rejected, rejected, and falsely accepted pipes per piece is obtained. All of the values are calculated for risks determined at the lower limit of the acceptance interval.

The conformance probability for model $M_{2}$ and $M_{4,}$ when T_L_ = 80 μm is significantly lower compared to models $M_{1}$ and $M_{3}$ and is equivalent to 0.7659. In these models, 76.59% of the measurements are in accordance with the standards and 23.41% of the measurements do not comply with the standards. It is notable that there are significantly more unfavorable results for the number of accepted measurements in the $M_{2}$ and $M_{4}$ models compared to models $M_{1}$ and $M_{3}$. The explanation for such differences stems from the difference between the values for the best estimation ӯ and the lower limit of the interval tolerance. For models $M_{2}$ and $M_{4}$, this difference is 12 μm, while for the $M_{1}$ and $M_{3\;}$, this difference is 22 μm.

In order to ensure better product quality and to avoid the risk of delivering pipes to the market with the thickness of the coating in the interval of non-allowed values [W_L_, T_L_], it is necessary to increase the thickness of the epoxy coating. This increases the conformance probability and the number of accepted products that meet the standards and reduces the risk to consumers and the risk to producers.

For this purpose, in this study, the value of the best estimation, i.e., the thickness of coating ӯ, was determined so that 95.45% of all of the measurements are within the interval $\langle ӯ-2u_{0},\;ӯ+2u_{0}\rangle$. This is the rule of two-sigma. It is also valid that T_L_ = $ӯ-2u_{0}.$ In this case, for models $M_{1}$ and $M_{3},$ the best estimation ӯ = 102 μm, and for models $M_{2}$ and $M_{4}$ it holds that the best estimation is ӯ = 112 μm. Now, the new models were defined as: ${M_{1}}^{\prime }\left(70,\;102,\;16,\;2\right),{M_{2}}^{\prime }\left(80,\;112,\;16,\;2\right),{{\;M}_{3}}^{\prime }\left(70,\;102,\;16,\;4\right)$, and ${M_{4}}^{\prime }\left(80,\;112,\;16,\;4\right).$

In the ${M_{1}}^{\prime }$ model, the lowest consumer risk, determined at the lower limit of the acceptance interval, is R_C_ = 0.005%. At the lower limit of the acceptance interval, the highest producer’s risk was determined, and it amounts to R_P_ = 1.7%. If we want to ensure that the values for the thickness of the epoxy coating are outside the interval [W_L_, T_L_], then the consumer’s risk in model ${M_{i}}^{\prime },\;i=1,2,3,4$ for r = −1, denoted by R_−1_, must be less than the consumer’s risk in model $M_{i},\;i=1,2,3,4$ for r = 0. The risk obtained for r = 0, i.e., the risk that is obtained when T_L_ = A_L_, is called the shared risk. This risk is denoted by R_0_. In model $M_{1}$, shared risk, i.e., the consumer’s risk at the lower limit of the tolerance interval is equivalent to R_0_ = 0.75%, and in the ${M_{1}}^{\prime }$ model, the risk R_−1_ = 0.76%. This means that it may happen that the thickness of the coating is within the interval of non-allowed values, and for this model, the value ӯ needs to be increased. It is enough to put that ӯ = 103 μm. Then, R_−1_ = 0.63% and the coating thicknesses are within the allowed values. The ӯ = 103 μm risks at the lower limit of the acceptance interval are now R_C_ = 0.004% and R_P_ = 1.47%. In the ${M_{2}}^{\prime }$ model, the minimum consumer’s risk, at the lower limit of the acceptance interval is R_C_ = 0.005%, and the highest risk to the producer is R_P_ = 1.72%. Since R_0_ = 1.6% and R_−1_ = 0.79%, the thicknesses of the coating are within the allowed values. In the case of model ${M_{3}}^{\prime }$, the lowest consumer’s risk is R_C_ = 0.008%, and the highest producer’s risk is R_P_ = 5%. Here, R_−1_ = 1.07% and R_0_ = 1.31%, so the coating thicknesses are within the allowed values. For the ${M_{4}}^{\prime }$ model, the lowest consumer’s risk is R_C_ = 0.008% and R_P_ = 5.02%. The coating thicknesses are within the allowed values because R_−1_ = 1.12% and R_0_ = 2.96%. The values $u_{0},$ $u_{m}$, and T_L_ remained unchanged.

**Figure 3:**
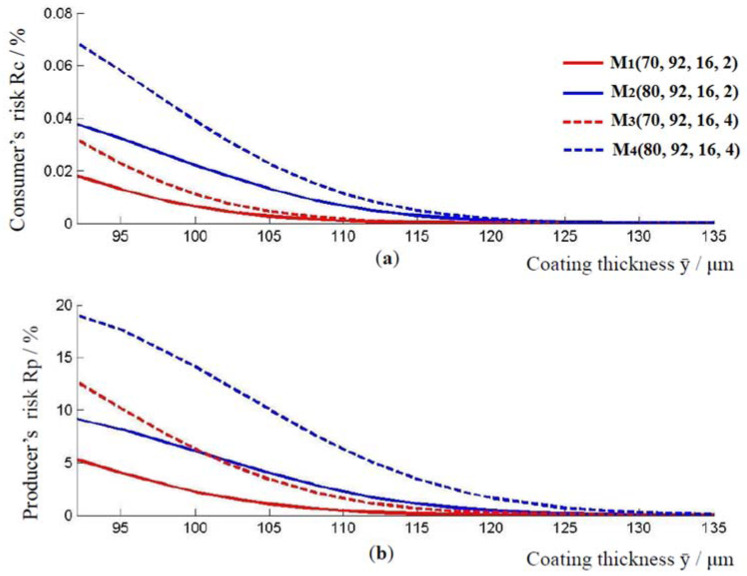
The relationship between the applied thickness of the coating and the consumer’s and producer’s risk. (**a**) Change in consumer risk with increasing coating thickness, and (**b**) change in consumer risk with increasing coating thickness, for models $M_{1},{\;M}_{2},{\;M}_{3}\text{ and}{\;M}_{4}$.

The relationship between the applied thickness of the coating and the consumer’s and producer’s risk for models $M_{1}$, $M_{2}$, $M_{3}$, and $M_{4}$, is shown in Figure 3. From this graph, for a given coating thickness ӯ≥92 μm, the values for the consumer’s risk and the producer’s risk can be determined. With an increase in coating thickness, the producer’s risk and the consumer’s risk decrease in all of the models. As before, model $M_{3}$, which has a higher standard measurement uncertainty compared to model $M_{1}$, has a higher producer’s and consumer’s risk. The same applies to models $M_{2}$ and $M_{4}$. By comparing the models $M_{1}$ and $M_{2}$, or models $M_{3}$ and $M_{4}$, in Figure 3, it is possible to observe how the value of consumer’s and producer’s risk changes when the lower limit of the tolerance interval changes from 70 μm to 80 μm. Model $M_{2}$ with a larger lower limit of the tolerance interval has a higher producer and consumer risk compared to model $M_{1}$ for the coating thickness ӯ≥92 μm. In addition, $M_{4}$ model, which has a higher lower limit of the tolerance interval, has a higher consumer’s and producer’s risk compared to model $M_{3}$.

With increasing the thickness of the epoxy coating, conformance probability also increases. For models ${M_{1}}^{\prime }$ and ${M_{3}}^{\prime }$, the conformance probability is 0.9860. This means that 98.6% of the measurements are conformed and 1.4% of the measurements are non-conformed. For models ${M_{2}}^{\prime }$ and ${M_{4}}^{\prime }$, the conformance probability is 0.9852, so 98.52% of the measurements are conformed and 1.48% of the measurements are non-conformed. The results for the percentage of accepted, rejected, falsely accepted, and falsely rejected measurements for models ${M_{1}}^{\prime },$ ${M_{2}}^{\prime },$ ${M_{3}}^{\prime }$ and ${M_{4}}^{\prime }$ are given in Table 1.

### 3.2. Pipe Junction

**Figure 4:**
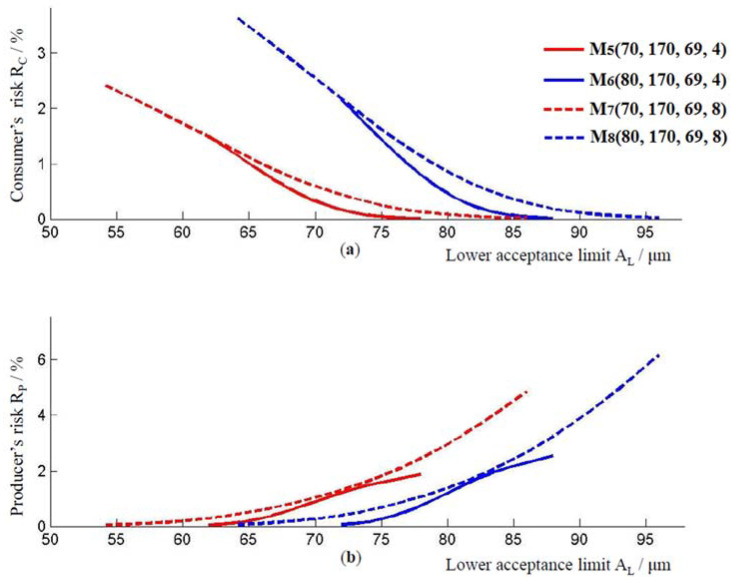
(**a**) Consumer’s risk and (**b**) producer’s risk, models $M_{5},{\;M}_{6},{\;M}_{7}\text{ and}{\;M}_{8}$.

At the thickness of the epoxy coating on the outside of the pipe, at the junction of two pipes, models $M_{5}\left(70,\;170,\;69,\;4\right),{\;M}_{6}\left(80,\;170,\;69,\;4\right),{\;M}_{7}\left(70,\;170,\;69,\;8\right),$ and model $M_{8}\left(80,\;170,\;69,\;8\right)$ were observed. Their associated models, where the value of ӯ is increased by applying the two-sigma rule are:${M_{5}}^{\prime }\left(70,\;208,\;69,\;4\right),$ ${M_{6}}^{\prime }\left(80,\;218,\;69,\;4\right),\;$ ${M_{7}}^{\prime }\left(70,\;208,\;69,\;8\right)$ and ${M_{8}}^{\prime }\left(80,\;218,\;69,\;8\right).$ The coating thickness ranges for models $M_{i}$ and ${M_{i}}^{\prime },\;i=5,6,7,8$ are [62, 78] μm, [72, 88] μm, [54, 86] μm, and [64, 96] μm, respectively. As with the previous models, the lowest consumer’s risk and the highest producer’s risk are achieved for the value of the lower limit of the acceptance interval. In model $M_{5}$, the lowest consumer’s risk, at the lower limit of the acceptance interval, is R_C_ = 0.007%, and the highest producer risk is R_P_ = 1.87%. For model $M_{6}$, these risks are R_C_ = 0.01% and R_P_ = 2.55%. The risks for the $M_{7}$ model are R_C_ = 0.014% and R_P_ = 4.84%. For the $M_{8}$ model, they are R_C_ = 0.019% and R_P_ = 6.17%, Figure 4.

Figure 4 also shows the impact of the standard measurement uncertainty $u_{m}$ on consumer and producer risk. Comparing models $M_{5}$ and $M_{7}$, that is, models $M_{6}$ and $M_{8}$, it is evident that models with higher measurement uncertainty have a higher consumer and producer risk.

According to the conformance probability, there are 95.93% conformed and 4.04% non-conformed measurements for models $M_{5}$ and $M_{7}.$ For models $M_{6}$ and $M_{8}$, there are 93.25% conformed and 6.75% non-conformed measurements. The results for the percentage of accepted, rejected, falsely accepted, and falsely rejected measurements are shown in Table 3.

For models where the thickness of the coating was increased, the following results were obtained. In the ${M_{5}}^{\prime }$ model, the minimum consumer’s risk, at the lower limit of the acceptance interval, is R_C_ = 0.001%, and the highest producer’s risk is R_P_ = 0.31%. For this model, R_−1_ = 0.20% which is less than R_0_ = 0.33% and thus ensures that the thickness of the epoxy coating is within the allowed values. For the ${M_{6}}^{\prime }$ model, the consumer’s risk of R_C_ = 0.001% and the producer’s risk of R_P_ = 0.34% were obtained. Since R_−1_ = 0.23% and R_0_ = 0.46%, the thickness of the coating are within the allowed values. For the ${M_{7}}^{\prime }$ model, it holds that R_C_ = 0.002% and R_P_ = 1.1%. The results obtained show that R_−1_ = 0.29% and R_0_ = 0.59%; these coating thicknesses are within the allowed values. The risks for the ${M_{8}}^{\prime }$ model are R_C_ = 0.002% and R_P_ = 1.15%. The coating thicknesses are within the allowed values because R_−1_ = 0.33% and R_0_ = 0.86%.

**Figure 5:**
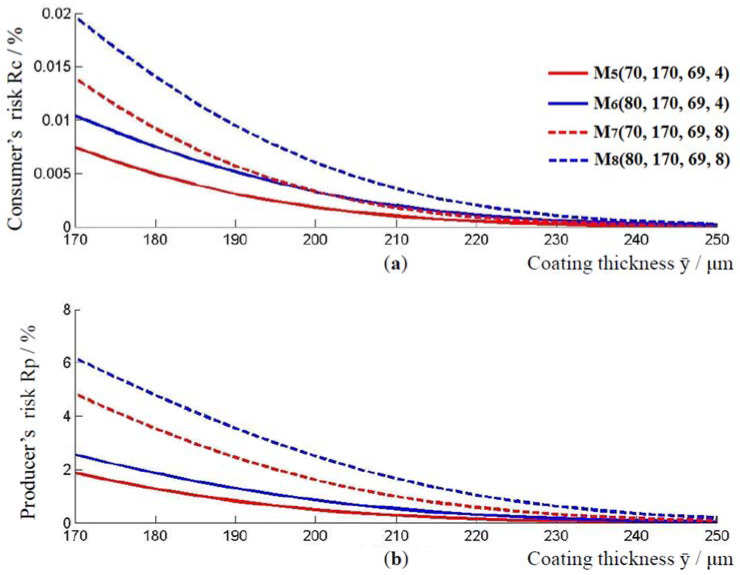
Relationship between the applied thickness of the coating and the consumer’s and producer’s risk. (**a**) Change in consumer risk with increasing coating thickness, and (**b**) change in consumer risk with increasing coating thickness, for models $M_{5},{\;M}_{6},{\;M}_{7}\text{ and}{\;M}_{8}$.

As before, with an increase in the thickness of the coating layer, the producer’s risk and the consumer’s risk decrease, Figure 5. Model $M_{7}$, with a higher standard measurement uncertainty compared to model $M_{5}$, has a higher producer’s and consumer’s risk, for the coating thickness ӯ≥170 μm. The same applies to the models $M_{6}$ and $M_{8}$. By comparing the curves for models $M_{5}$ and $M_{6}$, or models $M_{7}$ and $M_{8}$, it could be observed that the values of consumer’s and producer’s risk increase when the lower limit of the tolerance interval changes from 70 μm to 80 μm.

### 3.3. Common Risk

Within the production process, a pipe is considered conforming if both the thickness of the coating on the smooth outer area of the pipe and the thickness of the coating on the coupler are acceptable. A pipe is considered nonconforming if both the thickness of the coating on the outer area of the pipe and the thickness of the coating on the coupler are rejected. Furthermore, the pipe is nonconforming if only one area has an acceptable coating thickness while the other one does not. A pipe is considered nonconforming when either of the coatings has a thickness that is falsely accepted or falsely rejected, even when the other coating has a thickness that is accepted. Based on the values shown in Table 2 and Table 3, it is possible to calculate the percentage of conforming pipes. Results for all of the possible combinations of models are shown in Table 4.

The percentage of rejected pipes that are not in accordance with the given norms can be determined by subtracting the values given in Table 4 from 100.

The best combination of models, which would provide the largest number of conforming pipes, is the combination of model $M_{1}\prime \left(70,\;102,\;16,\;2\right)$ and model $M_{5}\prime \left(70,\;208,\;69,\;4\right)$. It should be noted that these are the models that simultaneously have the smallest value of the lower limit of the tolerance interval the largest coating thickness, and the smallest standard measurement uncertainty. For this combination, the percentage of conforming pipes is 96.17%. The smallest percentage of conforming pipes, 50.13%, can be achieved by combining models $M_{4}\left(80,\;92,\;16,\;4\right)$ and $M_{8}\left(80,\;170,\;69,\;8\right).$ These are the models that at the same time have the highest value of the lower limit of the tolerance interval, the lowest value of the layer thicknessm and the highest value of measurement uncertainty.

## 4. Discussion

The paper presents the conformity assessment process of the epoxy coating thickness applied on water pipes taking account of the value of the standard deviation u_0_ and the standard measurement uncertainty $u_{m}$ which characterize the quality of the coating application process and the quality of the measurement system. The models for the evaluation of the conformity with the condition of the lesser standard measurement uncertainty of the measurement system $u_{m}$ have been simulated. The models for the straight and junction sections of the pipe have been tested. The analysis was carried out for the recommended epoxy coating thickness, in accordance with the EN 877 standard, in the amount of T_L_ = 70 μm and T_L_ = 80 μm.

All of the mentioned quantities significantly affect the assessment of consumer risk and producer risk. First the influence of the value of the lower limit of the tolerance interval given by the norm on the risk assessment is considered. According to (5), conformance probability (specific risk) is defined as the area under the subintegral function within the limits set by the tolerance interval. For models with a lower value of the lower limit of the tolerance interval, the conformance probability has a higher value, if the upper limits of the tolerance interval coincide. The value for conformance probability of models $M_{1}$ and $M_{3}$ is higher compared to models $M_{2}$ and $M_{4}$ on the straight surface of the pipe. The same applies to the models at the pipe junction. The conformance probability of models $M_{5}$ and $M_{7}$ is higher than the conformance probability for models $M_{6}$ and $M_{8}$. The difference is not negligible. On the straight part of the pipe, in the range from 70 μm do 80 μm, the conformance probability decreased by as much as 17%. At the pipe junction, on the range from 70 μm to 80 μm, the conformance probability decreases by 3%.

The difference in conformance probability values for models on the outer straight part of the pipe and on the pipe junction is due to the thickness of the epoxy coating layer. Due to the requirements of production processes, thicker layers of epoxy coating are applied to the pipe junction, which affects the conformance probability. Therefore, the conformance probability can be increased if a thicker coating layer is applied. This can be seen by comparing the models $M_{i},\;i=1,\ldots ,8$ and $M_{i}^{\prime },\;i=1,\ldots ,8,$ shown in Table 2 and Table 3. A higher value for conformance probability implies a higher number of conformed pipes compared to the number of non-conformed pipes. Therefore, a greater number of correct pipes can be obtained by combining models with a smaller lower limit of the acceptance interval and a larger thickness of the epoxy coating layer, as shown in Table 4.

As the thickness of the epoxy coating layer increases, the consumer’s risk and the producer’s risk decrease, as shown in Figure 3 and Figure 5. It was shown in this research that the optimal value of the thickness of the epoxy coating layer is already achieved at the two-sigma distance from the lower limit of the tolerance interval.

The consumer’s risk and the producer’s risk directly affect the number of accepted pipes conformed to the specifications, and they respectively rejected pipes as non-conforming with specifications. When the consumer’s risk and the producer’s risk at the lower limit of the acceptance interval were compared, the consumer’s risks and producer’s risks are again smaller for the models tested for the lower limit of the recommended thickness of the epoxy coating layer. However, when comparing the consumer’s risk and producer’s risk in models with the same lower limit of tolerance interval T_L_, the same layer thickness ӯ, and the same standard deviation $u_{0},$ but with different standard measurement uncertainties $u_{m}$, then models with a smaller value of the standard measurement uncertainty $u_{m}$ have lower risks, as shown in Figure 2 and Figure 4. This speaks to the importance of measured uncertainty and its impact on risk assessment.

## 5. Conclusions

From the consumer’s point of view, the consumer wants a correct pipe whose service life will be in accordance with the prescribed norms. This can be ensured by increasing the thickness of the epoxy coating. Greater coating thickness certainly reduces the consumer’s risk but brings additional costs to the producer. The producer, on the other hand, wants to reduce costs and determine the optimal thickness of the coating. This mathematical model allows the producer to determine the optimal thickness of the epoxy coating based on the data obtained from the measurements and offers the consumer a product for which it the appropriate level of risk can be guaranteed. This risk assessment model is also applicable to other types of measurements.

Based on the analysis that has been conducted, the following conclusions have been reached:

1. The lesser value of the measurement uncertainty $u_{m}$ leads to a reduced probability that the rejected product is conforming. This benefits the producer. A larger value of standard uncertainty leads to a lager probability that the accepted product is non-conforming.
2. The lesser value of the standard measurement uncertainty $u_{m}$ benefits the consumers in that it protects them from non-conforming products, but it also benefits the producers in the sense that it reduces the likelihood of a wrongful rejection of the conforming product when global risk is calculated. The greater value of the standard measurement uncertainty um harms producers in the sense of the wrongful rejection of the conformity of the product and it also damages the consumer in the sense of the use of non-conforming products when calculating global risk.
3. Measurement uncertainty plays a significant role in the conformity assessment process, especially with decisions based on the results of those measurements that are close to the tolerance limit and may be inaccurate and may lead to unintended consequences.
4. In order for the likelihood of wrongful decisions being made to be contained within acceptable parameters, a consensus between producers and consumers is paramount. When estimating risk, it is essential to take stock of the measurement uncertainty of the measurement results.
5. The rule of divided risk can be proposed as a consensus between producers and consumers. When such a rule is deployed, both the producer and the consumer accept or reject a sample as conforming or non-conforming. As the very name suggests, divided risk says that by using this decision rule, both the producer and the consumer share the consequences for the wrongfully made decisions.

## Figures and Tables

**Table 1:** Coating thickness measurement results.

	Arithmetic Mean ӯ, μm	Standard Deviation $u_{0},$ μm	Standard Measurement Uncertainty $u_{m},$ μm
Straight part	92	16	4
Pipe junction	170	69	8

**Table 2:** Conformance probabilities for the straight, outer side of the pipes.

Model	p_c_	Accepted/%	Falsely Rejected/%	Rejected/%	Falsely Accepted/%
M_1_	0.9251	87.224	5.287	7.472	0.018
M_1′_	0.9860	96.900	1.7	1.395	0.005
M_2_	0.7659	67.175	9.145	23.372	0.038
M_2′_	0.9852	96.800	1.72	1.475	0.005
M_3_	0.9251	79.847	12.663	7.458	0.032
M_3′_	0.9860	93.600	5	1.392	0.008
M_4_	0.7659	57.565	19.025	23.341	0.069
M_4′_	0.9852	93.500	5.02	1.472	0.008

**Table 3:** Conformance probabilities for the pipe junction.

Model	p_c_	Accepted/%	Falsely Rejected/%	Rejected/%	Falsely Accepted/%
M_5_	0.9593	94.06	1.87	4.063	0.007
M_5′_	0.9956	99.25	0.31	0.439	0.001
M_6_	0.9325	90.7	2.55	6.74	0.01
M_6′_	0.9949	99.15	0.34	0.509	0.001
M_7_	0.9593	91.09	4.84	4.056	0.014
M_7′_	0.9956	98.46	1.1	0.438	0.002
M_8_	0.9325	87.08	6.17	6.731	0.019
M_8′_	0.9949	98.34	1.15	0.508	0.002

**Table 4:** Percentage of correct pipes.

Model	M_1_	M_1′_	M_2_	M_2′_	M_3_	M_3′_	M_4_	M_4′_
M_5_	82.04	91.14	63.18	91.05	75.10	88.04	54.15	87.95
M_5′_	86.57	96.17	66.67	96.07	79.25	92.90	57.13	92.80
M_6_	79.11	87.89	60.93	87.80	72.42	84.90	52.21	84.81
M_6′_	86.48	96.08	66.60	95.98	79.17	92.80	57.08	92.71
M_7_	79.45	88.27	61.19	81.18	72.73	85.26	52.44	85.17
M_7′_	85.88	95.41	66.14	95.30	78.62	92.16	56.68	92.06
M_8_	75.95	84.38	58.50	84.29	69.53	81.51	50.13	81.42
M_8′_	85.78	95.29	66.06	95.19	78.52	92.05	56.61	91.95

## Data Availability

Not applicable.
